# Inoculability of Phthisis

**Published:** 1869-04

**Authors:** 


### Inoculability of Phthisis.

M. Chauveau has succeeded in producing tuberculosis in three young heifers, by the administration *per orem* of tuberculous matter taken from the lungs of a phthisical cow. At the end of fifty-two days from the commencement of this experimental diet, the animals were killed, and the lungs, mesentery, and intestines presented marked evidences of tubercular disease. From these experiments he deduces the following conclusions:

"1st.—They place beyond all doubt the virulence and the contagious properties of tuberculosis, and demonstrate that the labors of M. Villemin upon the subject have not been recompensed as they deserve.

"2d.—The digestive tube in man constitutes, as in the bovine species, an avenue of contagion most appropriately arranged for the propagation of tuberculosis, and which may be much more frequently in operation than the pulmonary tract.

"3d.—If bovine tuberculosis belong to the same species as human tuberculosis, there exists in alimentation by butcher's meat, taken from phthisical animals, a permanent danger to public health, a danger to which the army and the poor classes are exposed, and against which it would be important to establish measures of sanitary police."

There are three questions left unsettled by M. Chauveau's experiments: firstly, whether three tuberculous individuals of a notoriously tuberculous species really "place beyond all doubt" the contagiousness of the disease; secondly, whether the malady is intercommunicable between heifers and human beings; and thirdly, whether the process of cooking "butcher's meat" may not obviate the "danger to the public health," or, in other words, whether Chauveau would not be contradicted by *veau chaud.*—*Med. Gazette.*

